# Reappraisal of the Roles of the Sonic Hedgehog Signaling Pathway in Hepatocellular Carcinoma

**DOI:** 10.3390/cancers16091739

**Published:** 2024-04-29

**Authors:** Kuo-Shyang Jeng, Chiung-Fang Chang, Yuk-Ming Tsang, I-Shyan Sheen, Chi-Juei Jeng

**Affiliations:** 1 Department of Surgery, Far Eastern Memorial Hospital, New Taipei City 220, Taiwan; 2 Department of Medical Research, Far Eastern Memorial Hospital, New Taipei City 220, Taiwan; changcf@mail.femh.org.tw; 3 Department of Imaging Medicine, Far Eastern Memorial Hospital, New Taipei City 220, Taiwan; mintsang@gmail.com; 4 Department of Gastroenterology & Hepatology, Linkou Chang Memorial Hospital, Chang Gung Medical Foundation, Taoyuan City 333, Taiwan; happy95kevin@gmail.com; 5 Graduate Institude of Clinical Medicine, National Taiwan University, College of Medicine, Taipei City 10617, Taiwan; b91401102@ntu.edu.tw

**Keywords:** Sonic hedgehog signaling pathway, hepatocellular carcinoma, cancer stem cells, recurrence, resistance

## Abstract

**Simple Summary:**

Hepatocellular carcinoma (HCC) remains one of the leading causes of cancer mortality. Treatment of HCC remains challenging, especially for those with advanced stages or those with postoperative recurrence. Molecular research of signaling pathways to afford new options of treatments is urgent. Moreover, among the various signaling pathways, the Sonic hedgehog (SHH) signaling pathway is implicated in multiple aspects of HCC, including cancer development, growth, invasiveness, recurrence, metastasis, the tumor microenvironment, and the maintenance of its cancer stem cells. The SHH signaling pathway also contributes to the resistance of HCC to chemotherapy, target therapy, and radiation therapy. This narrative review of the update studies reappraises the roles of SHH signaling in HCC. A deeper understanding of the SHH signaling pathway is crucial. It may provide more insights into the regulatory processes to establish novel treatments for HCC.

**Abstract:**

HCC remains one of the leading causes of cancer-related death globally. The main challenges in treatments of hepatocellular carcinoma (HCC) primarily arise from high rates of postoperative recurrence and the limited efficacy in treating advanced-stage patients. Various signaling pathways involved in HCC have been reported. Among them, the Sonic hedgehog (SHH) signaling pathway is crucial. The presence of SHH ligands is identified in approximately 60% of HCC tumor tissues, including tumor nests. PTCH-1 and GLI-1 are detected in more than half of HCC tissues, while GLI-2 is found in over 84% of HCC tissues. The SHH signaling pathway (including canonical and non-canonical) is involved in different aspects of HCC, including hepatocarcinogenesis, tumor growth, tumor invasiveness, progression, and migration. The SHH signaling pathway also contributes to recurrence, metastasis, modulation of the cancer microenvironment, and sustaining cancer stem cells. It also affects the resistance of HCC cells to chemotherapy, target therapy, and radiotherapy. Reappraisal of the roles of the SHH signaling pathway in HCC may trigger some novel therapies for HCC.

## 1. Introduction

HCC remains one of the leading causes of cancer-related death globally [1,2,3,4,5,6,7,8,9,10,11]. Recent epidemiological studies have observed a shift in the burden of HCC from regions with low to moderate sociodemographic indexes to those with high sociodemographic indexes [1]. This relocation indicates a transition from viral etiologies to non-viral causes. The prevalence of hepatitis B virus (HBV) and hepatitis C virus (HCV) infections surpasses 50% in Asian and African countries [8]. Asian countries such as China, Korea, India, Japan, and Thailand exhibit a higher incidence of HBV-related HCC cases. On the other hand, China, Japan, and the United States show a greater prevalence of HCV-related HCC cases. The United States also demonstrates a higher proportion of alcohol-related HCC patients [9]. Nonalcoholic fatty liver disease (NAFLD) contributes to 1% to 38% of HCC burden in different countries [10]. Nonalcoholic steatohepatitis-related HCC patients are more prevalent in India, the United States, and Thailand [9]. Rumgay, H. et al. have projected an increase in the number of new HCC cases until the year 2040 [11].

A collaborative approach involving surgeons, oncologists, and radiologists is recommended for optimal management of HCC [12]. However, current treatment modalities, such as surgical resection, liver transplantation, radiofrequency ablation (RFA), transcatheter hepatic arterial chemoembolization (TACE), ethanol injection, targeted therapy, and immune therapies face various challenges. These challenges primarily arise from high rates of recurrence and limited efficacy in treating advanced-stage patients [3,4,5,6,7]. Therefore, there is an urgent need to extensively explore novel signaling pathways to identify potential therapeutic targets for HCC.

The Sonic hedgehog (SHH) signaling pathway has been recognized as a crucial factor in HCC among various signaling pathways [13,14,15]. The molecules involved in the SHH signaling pathway are believed to contribute to the process of carcinogenesis in patients with chronic liver injury. This pathway contributes to the progression of pre-cancerous lesions to malignant behavior [15,16,17,18,19,20,21]. Furthermore, the SHH signaling pathway has been implicated in various aspects of HCC, including cancer development, progression, invasiveness, recurrence, metastasis, tumor microenvironment, drug resistance (such as chemotherapy and targeted therapy), radiotherapy resistance, and maintenance of cancer stem cells. This article aims to provide an updated review of recent studies that explore the complex and pivotal roles attributed to the SHH signaling pathway in HCC. 

## 2. Sonic Hedgehog (SHH) Signaling Pathway 

The hedgehog (HH) signaling pathway plays a significant role in various cellular processes, including cell growth, cell differentiation, tissue patterning, embryonic development, organ homeostasis, tissue repair, and stem cell activities [13,14,15,16,17]. In normal tissues, this pathway remains inactive [13,14,15,16,17]. However, in cancerous organs, including the liver, aberrant activation of the HH signaling pathway has been observed [13,18,19,20,21].

**Figure 1:**
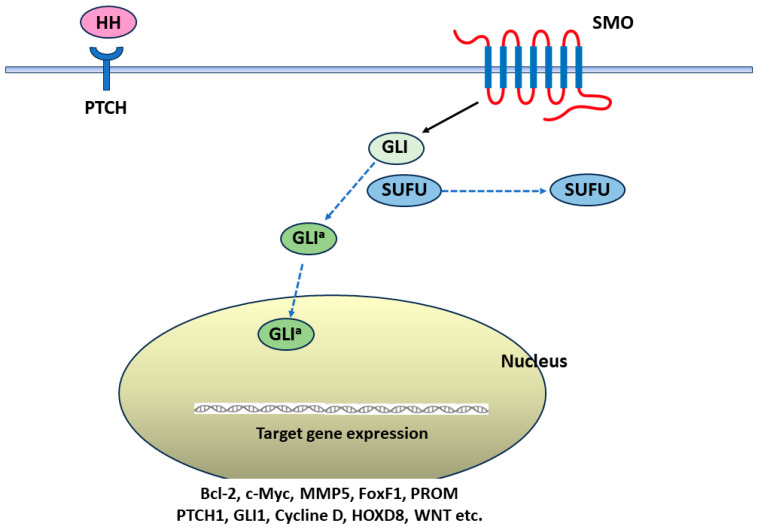
Canonical hedgehog signaling pathway acts after the hedgehog (HH) ligands join the Patched-1 (PTCH-1) to release Smoothened (SMO) and enhance the translocation of glioma-associated oncogene (GLI) into the nucleus. The suppressor of fused protein (SUFU) is liberated from the binding of GLI. Then, GLI activator form (GLI^a^) manipulates the target gene expressions (B-cell lymphoma-2 (Bcl 2)) gene for cell survival; c-Myc gene for cell proliferation; matrix metalloproteinase (MMP) gene for migration and invasion; Forkhead Box-F 1 (FoxF 1) gene for angiogenesis; prominin (PROM) for cancer stem cells; homeobox D8 (HOXD8; Homo sapiens) for cell proliferation, migration and invasion; etc.

The canonical HH signaling pathway involves several molecular components, including HH ligands (such as Sonic hedgehog (SHH), Indian hedgehog (IHH), and Desert hedgehog (DHH)), patched (PTCH-1 and PTCH-2) receptors, Smoothened (SMO) receptors, and glioma-associated oncogene (GLI) transcription factors (including GLI-1, GLI-2, and GLI-3) [13,14,15,16,17,18,19]. The HH signaling pathway orchestrates various cellular processes by initiating a series of events triggered by the binding of HH ligands to their receptor PTCH-1 [13,14,15,16,17,18,19,20,21,22,23]. This binding event relieves PTCH-1 inhibition on the G-protein-coupled receptor (GPCR)-like signal transducer SMO. Consequently, activated SMO prevents the separation of GLI-2/3 transcription factors [13,14,15,16,17,18,19,20,21,22,23]. Subsequently, GLI proteins translocate to the nucleus and bind to specific gene promoters, activating the transcription of target genes such as B-cell lymphoma-2 (Bcl-2) (antiapoptotic), cyclins (cell proliferation), and SNAIL (inducing epithelial–mesenchymal transition—EMT) [13,14,15,16,17,18,19,20,21,22,23,24]. Additionally, the SHH signaling pathway is negatively modulated by the suppressor of fused protein (SUFU). However, mutations in SUFU can lead to aberrant activation of the SHH pathway [18]. Activation of SMO occurs after SHH ligands bind to PTCH, relieving the suppression on SMO [13,14,15,16,17,18,19,20,21,22,23,24,25]. Activated SMO translocates to the plasma membrane, transmitting signals to the nucleus through GLI proteins to activate target genes [13,14,15,16,17,18,19,20,21,22,23,24,25]. This ultimately influences cell invasion, cell growth, and stem cell activity in cancer (Figure 1).

**Figure 2:**
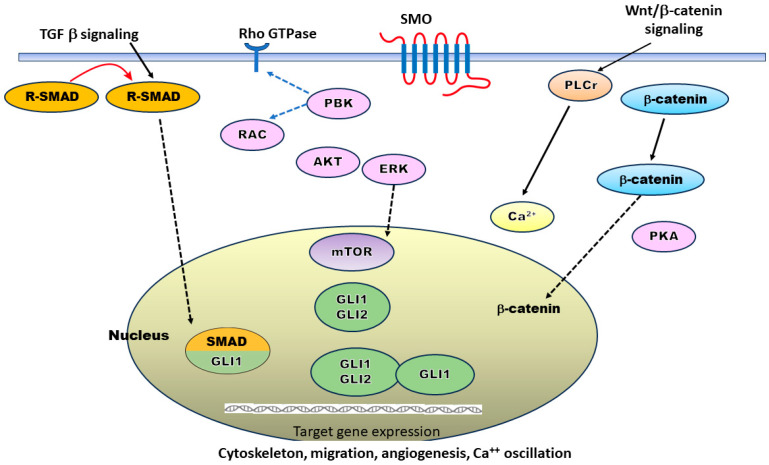
Non-canonical signaling pathway escapes Patched-1 (PTCH-1), Smoothened (SMO), and glioma-associated oncogene (GLI), which are activated via other signaling pathways such as guanosine triphosphate hydrolases (GTPase) or Rhodopsin (Rho), phosphoinositide 3-kinase mammalian target of rapamycin inhibitor (PI3K/mTOR), protein kinase (PKA), etc., to allow the target gene expressions. PKA phosphorylates the C-terminus of SMO at three sites (transcription factor mothers against decapentaplegic protein—SMAD). Phosphoinositide 3-kinase (PI3K) is activated via AKT (protein kinase B, PKB), mammalian target of rapamycin inhibitor (mTOR), etc. PI3K interacts with Rho A and Rac for the cytoskeleton, and the pleiotropic regular of extracellular virulence factor gene (PLCr) acts on sustained calcium 2+ (Ca 2+) flux. Thus, the pathway adjusts cytoskeleton, cell migration, angiogenesis, calcium oscillation, etc.

In addition, there is an alternative non-canonical pathway that operates when the canonical HH pathway fails to activate [13,15,18]. The non-canonical SHH signaling pathway involves the activation of SMO or GLI through alternative pathways, such as the mammalian target of rapamycin-protein kinase B (mTOR-Akt), guanosine triphosphate hydrolases (GTPase), phosphoinositide 3-kinase (PI3K)/mTOR, protein kinase A (PKA), or Ras homology (Rho) pathways [13,14,15,16,17,18,19,20,21,22,23,24,25] (Figure 2). Indeed, the SHH signaling pathway actively interacts with multiple other signaling pathways, resulting in intricate crosstalk [15,18,24,25,26].

## 3. Sonic Hedgehog (SHH) Signaling Pathway in Hepatocellular Carcinoma (HCC)

**Figure 3:**
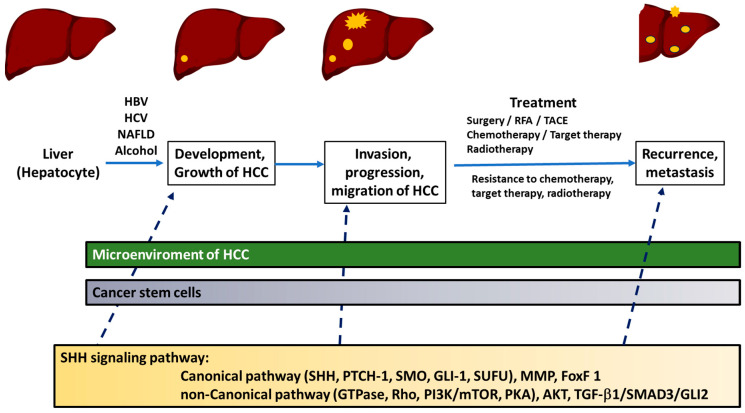
The illustration shows the roles of Sonic hedgehog signaling pathway in different stages of hepatocellular carcinoma. RFA: radiofrequency ablation; TACE: transhepatic artery chemoembolization; SHH: Sonic hedgehog; PTCH-1: Patched-1; SMO: Smoothened; GLI: glioma-associated oncogene; SUFU: the suppressor of fused protein; MMP: matrix metalloproteinase; FoxF 1: Forkhead Box-F 1; GTPase: guanosine triphosphate hydrolases; Rho: Rhodopsin; PI3K: phosphoinositide 3-kinase; mTOR: mammalian target of rapamycin inhibitor; PKA: protein kinase; AKT: protein kinase B, PKB; TGF: transforming growth factor.

The presence of SHH ligands has indeed been identified in approximately 60% of HCC tumor tissues, including tumor nests [27,28]. Moreover, PTCH-1 and GLI-1 have been detected in more than half of HCC tissues [24,29], while GLI-2 is found in over 84% of HCC tissues [30]. The SHH signaling pathway has been emphasized and extensively studied in recent years due to its involvement in different aspects of HCC. It would be informative to review the specific roles of the SHH signaling pathway in HCC across various aspects, as mentioned in Table 1 and Figure 3.

### 3.1. The Role of the SHH Signaling Pathway in the Development and Growth of HCC

Among the various causes of HCC, chronic infection with HBV or HCV and NAFLD have been found to correlate with activation of the SHH signaling pathway [31,32,33,34]. One potential mechanism involves the transformed HBx protein (a product of the HBV gene), which can induce activation of the SHH signaling pathway [31,32]. In the context of HBV or HCV infection, the presence of these viruses can enhance the expression of HH ligands in hepatocytes, thereby expanding the population of cells responsive to HH signaling. HH ligands can bind to PTCH receptors on liver stellate cells and liver sinusoidal endothelial cells, triggering processes such as liver fibrosis, angiogenesis, and ultimately liver carcinogenesis [32,33].

In-depth research utilizing an HBx transgenic mouse model has provided further support for the involvement of the activated SHH pathway in the development of liver pathologies and hepatocarcinogenesis. Specifically, the activated SHH pathway has been shown to trigger steatosis, liver fibrosis, dysplastic nodules, and induce hepatocarcinogenesis in this mouse model [31]. In the context of NAFLD, hepatocytes that have been damaged by lipotoxicity can produce HH ligands, which in turn contribute to the activation of HH-responsive cells expressing GLI-2. This activation of the SHH pathway can then lead to liver fibrosis [31]. Notably, the SMO inhibitor Vismodegib has demonstrated efficacy in inhibiting the growth of HCC in HBx transgenic mice, effectively suppressing HBx-positive human HCC xenograft tumors [31]. Other SMO inhibitors, such as GDC-0449 or LED 225, have shown potential in ameliorating liver inflammation in mice with NAFLD [34]. It has been reported by Cai et al. that during hepatocarcinogenesis, the activated SHH signaling pathway and increased SMO levels may enhance cell proliferation by facilitating the G2/M transition. This phenomenon is believed to occur through the overexpression of cyclin-dependent kinase 1 (CDK1) and cyclin B1 [35]. Moreover, SMO has been implicated in the repair of adult liver tissue and may be upregulated in primary hepatocytes after Fas-induced injury [18,34]. Additionally, SMO may play a role in promoting epithelial–mesenchymal transition (EMT) during early hepatocarcinogenesis [15,34,35,36].

It has been proposed that overexpression of SMO-mediated c-Myc could contribute to the development of hepatocarcinogenesis [20,33]. Moreover, mutations in SMO, such as the C-terminal lysine mutation (K575M), have been implicated in allowing SMO to evade PTCH suppression and activate downstream signaling [18].

Numerous studies provide additional support for the crucial role of the SHH signaling pathway and SMO activation. Philips et al. demonstrated a link between the SHH signaling pathway and the development of pre-cancerous lesions with liver fibrosis and hepatocarcinogenesis. They utilized GDC-0449, an SMO inhibitor, and observed a decrease in GLI-1 and GLI-2 levels along with an improvement in liver fibrosis [37]. Similarly, Pinter et al. found that GDC-0449, when used in an orthotopic HCC model, led to a reduction in angiogenesis [38]. Jeng et al. utilized cyclopamine, another SMO inhibitor, in a mouse hepatoma xenograft model and observed a reduction in tumor size, SHH mRNA, and GLI-1 mRNA levels [39]. 

Dysregulation of the hedgehog-Forkhead box M-targeting protein for the xenopus kinase-loke protein 2 (Hh-FOXM1-TPX2) signaling pathway, as observed by Wang et al., has been found to contribute to the cellular proliferation of HCC [40]. Antagonists of the SHH pathway signaling, such as cyclopamine and GANT 61, have shown the ability to downregulate TPX2, suggesting that SHH signaling enhances cellular proliferation in HCC through the regulation of the FOXM1-TPX2 signaling pathway [40].

In a study by Tripathy et al., the expression levels of SHH were examined at different stages of HCC using a N-nitrosodiethylamine (DEN) plus carbon tetrachloride (CCl4) model in male Wistar rats [96]. They observed an increase in liver lipidosis and fibrosis associated with an increase in SHH expression [96]. The activated SHH pathway has been recognized for its role in promoting fibrosis and lipogenesis in various malignancies [97,98]. Additionally, a high level of adiponectin has been implicated in exacerbating the clinicopathology of HCC [99,100]. 

Recently, Ding et al. elucidated the role of non-canonical hedgehog activation via transforming growth factor β 1/SMAD 3 (TGF-β-1/SMAD 3/GLI-2) in the progression of HCC [41]. Zhu et al. reported that high expression of TGF-β existed in chronic liver injury as a precarcinogenic niche to activate hepatic stellate cells to trigger liver fibrosis [101]. 

To summarize, SMO, GLI, Hh-FOXM1-TPX2, and non-canonical hedgehog activation via TGF-β-1/SMAD 3 are involved in hepatocarcinogenesis and tumor growth. 

### 3.2. The Role of the SHH Signaling Pathway in the Invasiveness, Progression, and Migration of HCC

There is strong support for the significance of the SHH signaling pathway in determining the development of HCC beyond its initial formation. The invasiveness of HCC cells is likely facilitated by the activation of the non-canonical SHH signaling pathway and the induction of oxidative stress EMT due to hypoxia [42]. Tian suggests that the SHH signaling pathway contributes to the invasiveness of hepatoma cells, while Fan’s findings indicate that abnormal SHH signaling has an impact on the invasiveness of HCC cells [36,43]. Che’s research reveals that activation of the SHH signaling pathway is positively correlated with tumor size, capsular invasion, and vascular permeation in HCC [44,45]. Jeng et al. conducted a study demonstrating higher expressions of SHH, PTCH-1, and GLI-1 in HCC tissues compared to adjacent non-cancerous liver tissue. Additionally, they found that high expression of SMO, along with a higher ratio of SMO mRNA to PTCH mRNA, significantly correlated with tumor size [29]. Ding’s findings indicated that aberrant activation of the SHH signaling pathway is associated with poor differentiation of HCC cells [46]. Jeng also found that overexpression of PTCH-1 mRNA correlates with the invasive characteristics of HCC, such as higher AFP value, larger size, vascular invasion, and tumor-node-metastasis stage (TNM stage) [29]. Tumor size has been consistently associated with portal venous invasion in HCC [29]. Chen’s research suggests that SHH is linked to capsular integrity, portal vein invasion, and distant metastasis [47]. Furthermore, GLI-1 has been found to correlate with tumor size, cellular differentiation, capsular integrity, and portal vein invasion [47]. Additional evidence supporting these findings is provided by Li Hy, who reported that elevated levels of SHH indicate portal vein invasion in HCC [48].

Aberrant activation of the SHH pathway leads to the nuclear entry of the transcription factor GLI-1, where it binds to the promoter site of target genes, thereby enhancing the biological behaviors of cancer cells [15,18,40]. Chen’s research indicates that the activated SHH pathway can trigger the focal adhesion kinase (FAK)/PI3K/AKT signaling pathway, leading to the upregulation of matrix metalloproteinase-2 (MMP-2) and matrix metalloproteinase-9 (MMP-9), thereby enhancing the invasion and migration of HCC cells [49]. Wang et al. propose that SHH signaling affects MMP-2 and MMP-9, contributing to the invasion and migration of HCC cells. They further suggest that bromodomain-containing protein 4 (BRD4) enhances this process, as inhibiting BRD4 reduces the activity of MMP-2 and MMP-9, thereby suppressing the progression and invasiveness of HCC cells [50].

Sicklick’s emphasis on the association between Rab 23 and the size of HCC is noteworthy [21]. Liu S.J.’s research suggests that downregulation of the Rab 23 gene may enhance apoptosis, decrease tumor size, reduce the proliferation rate, and decrease the levels of SHH-related proteins (SMO and GLI-1) in Hep 3B cells [51]. Moreover, Rab 23 actively promotes the migration of HCC cells and plays a crucial regulatory role in the SHH signaling pathway of HCC [51,52]. Some studies have highlighted the potential of the nuclear factor (erythroid-derived 2)-like 2 (NRF2) gene to bind to the SHH promoter, potentially activating the pathway and enhancing tumorigenicity [53,54,60,102,103,104]. Liu’s report suggests that chondroitin sulfate synthase 1 (CHSY1) can facilitate the growth, migration, invasiveness, EMT, and metastasis of HCC cells through the activation of the SHH signaling pathway [55]. Furthermore, their findings indicate that the use of Vismodegib can effectively suppress tumor invasion, migration, and metastasis to the lung in animal models [56].

Ding et al. reported that poor differentiation, poor prognosis, and higher recurrence are found in HCC with non-canonical GLI-2 activation [41]. They found a higher expression of TGF-β 1 and SMAD 3 in HCC specimens with GLI-2 positivity. The GLI-2 and pSMAD 3 may become cobound to the promoter sites of the genes contributing to carcinogenesis and progression [41]. In addition, from the poorly differentiated HCC, GLI-2 isoforms 6 and 7 were identified [41]. 

In summary, SHH signaling (SHH, PTCH-1, SMO, GLI-1), the FAK/PI3K/AKT signaling pathway, MMP-, MMP-9, Rab 23, CHSY 1, and activated GLI-2 by TGF-β 1/SMAD 3 participate in the invasiveness and progression of HCC.

### 3.3. The Role of SHH in the Recurrence and Metastasis of HCC 

The recurrence of HCC (after resection, transplantation, RFA, or TACE) is a complex and multi-step process [57]. Various factors contribute to recurrence, including the invasiveness of cancer cells, their detachment from the primary HCC site and entry into circulation, pre-existing microvascular invasion, the susceptibility of cancer cells to the liver remnant microenvironment, and the activation of the SHH signaling pathway. The metastatic process involves several sequential steps, such as disruption of the basement membrane, intravasation, migration, extravasation, and colonization in distant sites [22,48,52,55,56,57,58,59]. According to Ding et al., SHH signaling may play a role in each of these steps in the metastasis process [46]. 

There is compelling evidence showing that the gene expression of the SHH signaling pathway plays a significant role in the recurrence of HCC in patients [29]. High expression of molecules involved in the SHH signaling pathway, such as PTCH-1 and GLI-1, is strongly associated with an increased risk of recurrence after surgical resection [29]. Jeng et al., in their study, discovered that individuals with a higher ratio of GLI-1 mRNA in HCC tissue compared to non-cancerous tissue had a greater likelihood of recurrence and shorter survival durations [29]. Lin et al. emphasized that the presence of nuclear GLI-2 staining is significantly correlated with poorer differentiation of HCC tissues and tumor thrombosis of the portal vein [30]. Additionally, Wang et al. reported that SMO polymorphisms in liver transplant recipients are associated with a higher risk of HCC recurrence after liver transplantation [57].

Fan et al. reported that enhanced SHH activity and the aberrant emergence of a truncated GLI-1 (tGLI-1) variant lead to increased invasiveness and metastatic potential in cancer cells [43]. They also discovered that the SMO inhibitor (LDE225) curtailed the expression of matrix metalloproteinases (MMPs) and GLI-1/2, consequently reducing the invasiveness and metastatic propensity of hepatocellular carcinoma (HCC) cells [43]. Della Corte’s in vitro and in vivo inhibition studies indicated that the SHH signaling pathway plays a critical role [58]. Both GLI-1 and truncated GLI-1 are implicated in the metastasis of HCC cells [58]. Additionally, Dugum identified a link between the recurrence of HCC post-liver transplantation and elevated expression levels of SHH, PTCH, and GLI-1 proteins in both HCC and surrounding liver tissues [28].

Ding et al. found that a higher expression of SMAD protein was present in 87.5% of recurrent HCC with GLI-2 positivity [41]. This means that the non-canonical GLI-2 activation increased the tumor aggressiveness and the risk of HCC recurrence. From a transgenic HBV-related HCC mouse model, lung metastasis could be reduced after the inhibition of TGF-beta 1/SMAD 3 signaling [41].

In summary, the SHH signaling pathway (including SHH, PTCH-1, SMO, and GLI-1) and GLI-2 (driven by TGF-β 1/SMASD 3 signaling) affect the recurrence and metastasis of HCC. 

### 3.4. The Role of SHH in the Pathway of the HCC Microenvironment

Neophytou et al. emphasized the significant impact the tumor microenvironment (TME) has on the survival and progression of tumors [59]. The TME comprises various components including tumor-associated macrophages (TAMs) of the M2 type, cancer-associated fibroblasts (CAFs), the tumor extracellular matrix, lymphocytes, endothelial cells, various immune cells, and an array of cytokines [59].

Chung demonstrated that the hepatic expression of SHH might prompt the activation of hepatic stellate cells and induce the upregulation of certain fibrogenesis genes, thereby contributing to liver fibrosis and the onset of hepatocarcinogenesis [20]. Kwon observed that the activation of GLI, a key component of the SHH signaling pathway, could exacerbate liver inflammation through osteopontin-mediated macrophage activation. This process could accelerate the progression of NAFLD and increase the risk of developing HCC [34]. Liu et al. highlighted the role of a hypoxic microenvironment, commonly present in HCC, in enhancing intracellular reactive oxygen species (ROS)-mediated GLI-1-dependent EMT, which increases the invasiveness of HCC cells [42]. They employed N-acetylcysteine, an ROS inhibitor, to diminish ROS levels under hypoxia, thereby reducing GLI-1 expression, EMT progression, and the invasiveness of HCC cells [42].

Zhao J. discovered that the single peptide, CUB domain and EGF domain-containing 1 (SCUBE 1), was markedly abundant in the cancer-associated fibroblasts (CAFs) of HCC cells [61]. SCUBE 1 appears to augment the stemness features of HCC cells through the Sonic hedgehog (SHH)/GLI-1 pathway and further encourages malignant behaviors in tumors [61]. Additionally, Petty A.J. reported that SHH signaling could potentially promote TAMs, leading to an immunosuppressive environment [62]. 

We emphasized that GLI-1, ROS-mediated GLI-1-dependent EMT, and SCUBE 1 attend the microenvironment of HCC. 

### 3.5. The Role of the SHH Signaling Pathway in Chemoresistance, Resistance to Target Therapy, and Radioresistance

For patients with HCC who are ineligible for surgical resection or transplantation, alternative treatments such as RFA, TACE, or ethanol injection become essential. Additionally, for those who experience recurrence post-treatment, pharmacotherapy plays a critical role. This includes chemotherapy, which can be administered parenterally or through the TACE route, and targeted therapy. Radiotherapy may also be a viable option for certain patients.

Research into chemosensitivity, which influences the effectiveness of chemotherapy in treating cancer, has been relatively limited in the context of HCC [63,64,65,66,67,68]. Zhang et al. have shown that hypoxia, a prevalent feature of HCC, can heighten chemotherapy resistance by activating the SHH pathway [63].

Wang et al. reported that the downregulation of SHH signaling pathway activity can influence the apoptosis of Hep3B cells induced by 5-fluorouracil (5-FU) [68]. Their findings suggest that while activation of SHH can inhibit the motility of HCC cells, the overexpression of GLI-1 can counteract this effect, restoring cell viability, mobility, proliferation, and the capacity for migration in HCC cells [68].

Saikosaponin-d (Ssd) has been identified as capable of suppressing the malignant attributes of HCC cells while enhancing their chemosensitivity. It achieves this by increasing the expression of small ubiquitin-like modifier (SUMO) specific peptidase 5 (SENP5), which leads to the inhibition of GLI-1 SUMOylation. However, the exact mechanisms by which Ssd exerts its effects on HCC cells are not fully understood [63]. Furthermore, Chen has found that activation of epithelial–mesenchymal transition (EMT) and the SHH pathways contributes to chemoresistance in HCC. Adding to this, some have observed that the SHH signaling pathway can alter drug sensitivity in HCC through the ATP binding cassette subfamily C member 1 (ABCC1) transporter [65]. Ding also highlighted that the activation of certain target genes by GLI, namely ABCC1 and transporter associated with antigen processing 1 (TAP 1), are implicated in the drug resistance seen in HCC [66].

Additional research by Zhou revealed that the TAP 1 gene is among the targeted genes of GLI-1/2 and that both GLI-1 and TAP 1 play roles in determining the sensitivity of HCC cells to therapeutics such as sorafenib, doxorubicin, and cisplatin [67]. Additionally, Leung reported that resistance to sorafenib in HCC could be influenced by nuclear factor (erythroid-derived 2)-like 2 (NRF2), which is known to bind to the promoter of the SHH gene [54].

Wang’s studies utilizing patient-derived organoid (PDO) models of HCC demonstrated that CD44-positive HCC PDOs exhibited resistance to sorafenib [68]. However, the simultaneous application of sorafenib and GANT 61, an inhibitor of SHH signaling, was found to decrease cell viability and the malignant characteristics of HCC in both in vitro and in vivo settings. This suggests that GANT 61 may counteract the upregulation of CD44 and SHH signaling, hinting at its potential to reverse drug resistance [68].

Several investigators have highlighted the significant role of the NRF2 gene in chemotherapy resistance. For instance, the effectiveness of chemopreventive agents was reportedly compromised in NRF2-null mice, indicating the gene’s importance in withstanding chemotherapeutic insult [53]. Further reports have identified activation mutations of the NRF2 gene in approximately 6.4% of HCC samples, underscoring its role in liver cancer pathogenesis [53,54,60]. Leung et al. proposed that the upregulation of NRF2, induced by reactive oxygen species (ROS), may engender resistance to the anti-cancer drug sorafenib [54]. Moreover, NRF2 is believed to possibly interact with the promoter of the SHH gene, potentially amplifying the activation of the SHH signaling pathway and influencing drug sensitivity.

Ding et al. emphasized that SMAD 3-induced GLI-2 activation may transactivate downstream target genes affecting chemoresistance [41]. 

Another avenue of research suggests that the SHH protein, secreted by HCC cells, might negatively impact the sensitivity of these cells to radiation [69]. Activation of the SHH signaling cascade could lead to enhanced expression of SHH itself, as well as PTCH-1 and GLI-1. When soluble SHH peptides are introduced into a conditioned medium containing cultured human HCC cells, such as HA22T or Sk-Hep1 cells, the cells exhibit increased resistance to radiotherapy. In such a medium, counteracting SHH with specific antibodies, or disrupting GLI-1 through RNA interference (RNAi) or knockdown, can partially mitigate this radioresistant effect, hinting at the crucial role of SHH signaling in radiotherapy resistance. This is supported by evidence showing that SHH ligands inhibit the phosphorylation of checkpoint kinase 1 (Chk1) induced by radiation therapy [70]. Therefore, the mechanism behind SHH-induced radioresistance could involve the suppression of DNA damage repair. In their investigation using irradiated non-tumor cells, Leonard J.M. found that SHH signaling, when improperly activated, could exacerbate the effects of radiation on genomic instability and subsequent tumor progression [70]. This exacerbation is attributed to the disrupted interaction between Chk1 and Claspin, an adaptor protein critical for Chk1 activation. Thus, the aberrant activation of the SHH signaling pathway can lead to the impairment of Chk1 activation. The presence of SHH peptides may further influence the repair processes of radiation-induced DNA double-strand breaks. This suggests that targeting the SHH pathway could potentially enhance the effectiveness of radiotherapy by reducing radiation-induced DNA repair and tumor cell survival.

In related research, Tsai conducted studies using cyclopamine, a SHH pathway inhibitor, in combination with irradiation on human HCC cells (Huh7 and PLC/PRF/5) [71]. It was found that irradiation decreased GLI-1 expression and increased DNA double-strand breaks. In vivo experiments demonstrated that combining cyclopamine with radiotherapy, as opposed to radiotherapy alone, led to a significant decrease in orthotopic tumor size by about 67% (*p* < 0.05), indicating that the co-application of an SHH inhibitor with radiotherapy can boost the radiosensitivity of HCC cells [71]. Conversely, the researchers observed that the addition of an exogenous SHH ligand in vitro might diminish radiosensitivity and enhance radioresistance by primarily upregulating nuclear GLI-1 [71]. Hence, the inhibition of the SHH pathway with cyclopamine appears to augment radiosensitivity in both in vivo and in vitro settings. These findings underscore the potential of targeting the SHH signaling pathway as a strategy to improve the efficacy of radiation therapy in treating HCC.

In summary, GLI-1, SUMOylation, NRF2, TAP 1, and GLI-2 (induced by SMAD 3) participate in the resistance to chemotherapy and target therapy, whereas SHH, PTCH-1, GLI-1, Chk 1 are involved in radioresistance. 

### 3.6. The Role of the SHH Signaling Pathway in Cancer Stem Cells of HCC

Hepatic progenitor cells (HPCs) are located within the canals of Hering in an adult liver [72,73,74]. Their activation is often observed following liver injury [72,73]. HPCs are characterized by the co-expression of several stem cell surface markers such as EpCAM, CD133+, CD44+, NCAM, SOX9, SOX17, and CK18, as well as the presence of ligands for hedgehog (HH) signaling pathways like SHH and IHH [74]. When the liver is extensively injured, the prolonged activation of HH signaling can potentially lead to carcinogenesis and contribute to the maintenance of a stemness subpopulation within the liver. SHH signaling is crucial for sustaining the HPC population and for the maturation and differentiation of hepatic lineage cells [75,76].

Cancer stem cells (CSCs) bear a striking resemblance to normal stem cells and have the capacity to produce a diverse array of daughter cells with heightened levels of invasiveness and resistance to drugs [77,78,79,80,81,82,83,84,85,86]. These cells often exhibit traits consistent with the EMT phenotype, as evidenced by the expression of SNAIL, vimentin, TGF-beta, and components of the SHH pathway [77,78]. Notably, CSCs may be situated at the invasive edges of HCC nodules [58,59]. Additionally, HCC has been associated with a range of CSC markers [74,75,76,77,78,79,80,81,82,83,84,85,86,87,88,89,90,91,92,93,94,95,105]. Sukowati has highlighted the marked heterogeneity of HCC’s CSCs, a trait that likely complicates efforts to effectively inhibit these cells [84]. Wang S. has indicated that HH signaling is active in organoids or cells that are CD133+ and CD44+, both of which are derived from patients [68].

Among the diverse CSC populations in HCC, subpopulations marked by CD133+, CD44+, and CD90+ are particularly noteworthy for their resistance to sorafenib treatment, a common therapeutic agent used in managing HCC [68,83,84,85,86]. Wang S. has reported that Hh signaling is critical in sustaining the sorafenib-resistant phenotype seen in CD44+ cell populations [68]. This underscores the complexity of treating HCC due to the adaptability and resilience of these cancer stem cells.

Jeng reported that CD 133+ cells isolated from a mouse Hepa1-6-derived tumor showed a high expression of the components of the SHH signaling pathway [105]. They found that an activated SHH signaling pathway exists in CD133+ Hepa1-6 HCC cells in mice, with a downregulation of SHH mRNA and an upregulation of SMO mRNA [105]. CD133+ Hepa1-6 cells possess stem cell characteristics, including significantly higher colony proliferation and clonogenicity.

CD 90+ is another well-known CSC marker in HCC [95]. Multiple liver cancer cell lines and human HCC tissues have shown a strong correlation between CD90 expression and increased expression of GLI-1 and GLI-3. Analysis of cancer genome atlas (TCGA) data has revealed that individuals with CD90+ expression, along with GLI-1 and GLI-3, tend to have a relatively shortened overall survival rate [95]. Knocking down GLI-1/3 with siRNA can inhibit CD90+ CSCs in HCC, while SHH treatment can enhance their presence [95]. Additionally, Zhang et al. discovered that the stem cell properties of CD90+ liver cancer cells are regulated by the downstream signaling pathways of SHH/GLI and IL6/JAK2/STAT3, which can be modulated using the JAK2 inhibitor AZD1480 and interleukin-6 (IL6) neutralizing antibody [95].

Sari et al. also highlighted the significance of aberrant activation of the SHH signaling pathway in maintaining CSCs in various organs, including the liver [106]. Consequently, building upon the aforementioned research, they have suggested that targeting the SHH signaling pathway may lead to the eradication of CSCs.

Leung H.W. discovered that NRF2 may bind to the promoter region of SHH, potentially activating the SHH signaling pathway [54]. Furthermore, NRF2 might mediate the function of tumor-initiating cells (T-ICs) by upregulating SHH expression [54]. The canonical SHH pathway operates through the NRF2/SHH/GLI signaling axis to regulate T-IC phenotypes.

The main pathway and genes involved in cancer stem cells include the SHH/GLI-1 signaling pathway, IL6/JAK2/STAT 3 signaling pathway, GLI-1, GLI-3. NRF2, etc. 

### 3.7. Effects of the Inhibitiors of the SHH Signaling Pathway on HCC 

Given the involvement of the SHH signaling pathway in the development of HCC, inhibiting this pathway shows promise in positively impacting disease progression. Results from in vitro studies suggest that blocking the SHH signaling pathway can inhibit the growth and motility of HCC cells [39,107,108]. In a mouse model of fibrosis-associated HCC, treatment with an SHH inhibitor has shown potential in causing regression of advanced HCC [37]. Additionally, the administration of the SMO antagonist GDC-0449 appears to inhibit hepatocarcinogenesis in HBx transgenic mice [31].

Using a mouse model, Jeng found that both cyclopamine, an SMO inhibitor, and GDC-0449 can effectively inhibit SHH gene expression and inhibit the growth of HCC cells [38,108]. In particular, GDC-0449 has shown potential in suppressing cell migration, invasion, and distant metastasis to the lungs in CHSY1-induced HCC cells [55]. Cyclopamine, on the other hand, has demonstrated inhibitory effects on DNA synthesis, invasiveness, and migration of HCC cells [39]. Additionally, this drug downregulates Bcl-2, leading to the suppression of cell viability and induction of apoptosis in HCC cells [24]. Similarly, Cheng utilized KAAD-cyclopamine to inhibit the SHH signaling pathway, resulting in the inhibition of DNA synthesis, cell growth, invasiveness, and cell motility in HCC cells [36].

Sicklick’s research demonstrated that 3-keto-N-aminoethylcaproldihydrocinnamoyl cyclopamine (KAAD-cyclopamine) can suppress the SHH signaling pathway and inhibit the growth of Hep3B cells [21]. However, it is worth noting that Kim’s findings indicated that KAAD-cyclopamine was not effective in HCC cells with SMO mutations [109].

Combining bufalin, a topoisomerase II inhibitor, with other treatments has shown efficacy. Sheng’s study revealed that bufalin can suppress the invasion and metastasis of HCC cells through the SHH pathway [108]. They conducted a co-culture experiment with human hepatoma cells (HCC-LM3), bufalin, and SHH signaling pathway inhibitors (GANT61, cyclopamine) for 72 h. Bufalin appears to inhibit the epithelial–mesenchymal transition, angiogenesis, and extracellular matrix degradation in HCC cells through the SHH signaling pathway [108]. By inhibiting GLI-1 and GLI-3 in HCC cells, bufalin downregulates downstream target molecules (MMP-2, MMP-9, β-catenin, and VEGF) and upregulates E-cadherin expression, affecting GLI-3 expression. These findings suggest that combining bufalin with SHH signaling inhibitors may effectively suppress the invasiveness of HCC cells.

Sun et al. emphasized the role of the SHH gene in the metastatic potential of HCC [110]. They discovered that reducing the activity of the PI3K/Akt pathway could suppress HCC’s migration ability by inhibiting SHH-GLI-1 signaling. Furthermore, another recent study demonstrated that knockdown of the SHH gene impairs the function of the PI3K/Akt pathway, indicating the existence of a regulatory loop between SHH and PI3K/Akt. This suggests that inhibiting the SHH signaling pathway may reduce oncogenesis in HCC cells in vitro by suppressing the P13K/Akt pathway.

In liver specimens of patients with alcoholic hepatitis, upregulated SHH and GLI-2 proteins were detected [111]. Kumar et al. also found upregulation of miR-96 and SHH in ethanol-fed mice. Their study revealed that MDB5, an analog of GDC-0449 (an inhibitor of SHH), could reduce the activation of hepatic stellate cells and the expression of the GLI-1 gene. They proposed that MDB5, which suppresses the SHH signaling pathway, may attenuate the pathogenesis of alcohol-related liver disease in mice [111].

The Par-3 family cell polarity regulator (PARD3) is a cellular protein crucial for asymmetric cell division and polarized growth [112]. Wu et al. found that overexpression of PARD3 is associated with advanced tumor stage and a worse outcome [113]. Furthermore, overexpression of PARD3 enhanced tumorigenicity, tumor progression, and sustained the self-renewal ability of the CD133+ tumor-initiating cell population in HCC cells. In PARD3-overexpressing CD133+ tumor-initiating cells, activation of SHH signaling was observed. Inhibition of SHH signaling may reduce the tumorigenicity of PARD3-overexpressing CD133+ tumor-initiating cells both in vivo and in vitro. Wu et al. used berberine, a natural compound, to effectively suppress PARD3 expression in animal studies [113]. As mentioned earlier, the SHH/SMO signaling pathway plays a crucial role in the regulation of CSCs in HCC. Therefore, targeting the SHH/SMO signaling pathway could hold great promise for developing effective therapies against CSCs. By inhibiting this pathway, it may be possible to disrupt the self-renewal and tumorigenic properties of CSCs in HCC. Further research and exploration in this area of study could lead to the development of novel CSC-targeted therapies for HCC treatment.

Harada et al. studied the effect of GANT 61, which is a selective inhibitor of GLI-1 and GLI-2-mediated gene transactivation [114]. They used two undifferentiated hepatoma cell lines, HLE and HLF. They found that GANT 61 may inhibit cell viability and cell proliferation after treatment with 5-FU and mitomycin C. GANT 61 may target the non-canonical GLI signaling pathway. 

In summary, the inhibitors of the SHH signaling pathway include GDC-0449, cyclopamine, KADD-cyclopamine, GANT 61, suppression of the PI3K/Akt pathway, suppression of the PARD 3 signaling pathway, suppression of the TGF-beta 1/SMAD 3/GLI-2 signaling pathway, etc., whereas they are still limited in clinical applications. 

### 3.8. The Expressions of the SHH Signaling Pathway in Hepatocellular Carcinoma and Hepatocellular Adenoma (HCA)

As of now, there has been limited research regarding the differential expression of the SHH signaling pathway among the various histological types of HCC. The World Health Organization (WHO) classification categorizes HCC into different subtypes with distinct prognoses, including fibrolamellar, scirrhous, clear cell type, steatohepatitis, macrotrabecular mass (MTM), chromophobe, neutrophil-rich, and lymphocyte-rich [115,116]. It is worth noting that hepatocellular adenoma (HCA) has the potential to undergo malignant transformation into HCC. Different subtypes of HCA, such as hepatocyte-nuclear-factor-1 alpha mutated adenoma (H-HCA), inflammatory HCA (I-HCA), beta-catenin-mutated HCA (b-HCA), mixed inflammatory and beta-HCA (b-IHCA), HCA with Sonic hedgehog pathway activation (sh-HCA), and unclassified HCA (U-HCA), have been identified [117,118]. The presence of sh-HCA can be defined through magnetic resonance imaging (MRI), which reveals intralesional fluid-filled cavities [118]. Individuals with sh-HCA are often associated with obesity and have a higher incidence of hemorrhage [119]. Approximately 6% of patients belonging to this subgroup have been found to develop HCC [120].

Among the subtypes of HCA, sh-HCA remains the most important one in the development of HCC. 

## 4. Conclusions

The significance of the SHH signaling pathway in the context of HCC lies in its potential as a therapeutic target. This pathway plays a central role in various aspects of HCC, including hepatocarcinogenesis, tumor growth, invasiveness, recurrence, metastasis, modulation of the tumor microenvironment, and support of the maintenance of cancer stem cells. The non-canonical hedgehog activation via TGF-beta 1/SMAD 3 has to be emphasized. Furthermore, it has been implicated in HCC resistance to different treatment approaches. Targeting the SHH pathway for inhibition presents a promising new avenue to suppress the tumorigenic potential of HCC cells. Further research into the complex regulatory mechanisms of the SHH pathway holds the potential to unveil novel therapeutic strategies that can specifically target and suppress this pathway, ultimately leading to improved treatment options for HCC patients.

## Figures and Tables

**Table 1:** Summary of the roles of the Sonic hedgehog (SHH) signaling pathway in hepatocellular carcinoma (HCC).

M	Contributing Factors or Related Mechanisms	Biomarkers or Involved Factors	References
Development and growth	HBV, HCV, NAFLD, alcohol, liver cirrhosis, dysplastic nodules, angiogenesis	SHH, PTCH, SMO, CDK, cyclin β, Hh-FOXM1-TPX2, GLI-2(derived by TGF-beta 1/SMAN 3)	[18,31,32,33,34,35,36,37,38,39,40,41]
Invasiveness progression and migration	Hypoxia, tumor size, capsule, vascular permeation, portal venous invasion, poor differentiation	SHH, PTCH-1, GLI-1, FAK/P13K/AKT, MMP-2, MMP-9, CHSY 1, GLI-2 (derived by TGF-beta 1/SMAD 3)	[29,36,41,42,43,44,45,46,47,48,49,50,51,52]
Recurrence and metastasis	Basement membrane disruption, detachment of cancer cells entering circulation, intravasation, extravasation	SHH, PTCH-1, SMO, GLI-1, MMPs, GLI-2 (derived by TGF-β 1/SMAD 3)	[28,29,30,41,43,46,53,54,55,56,57,58]
Microenvironment of HCC	Hepatic stellate cells, TAMs, extracellular matrix lymphocytes, endothelial cells, immune cells	Fibrogenesis genes, ROS, EMT, SCUBE 1, cytokines	[34,43,57,58,59]
Resistance to chemotherapy, target therapy, and radiotherapy	Hypoxia, CD 44 positive HCC cells	SHH, PTCH-1, GLI-1, SUMO, ABCC 1, TAP 1, NRF 2, ChK 1, Claspin, GLI-2(derived by TGF-beta 1/SMAN 3)	[41,49,60,61,62,63,64,65,66,67,68,69]
Cancer stem cells maintenance	EpCAM, CD 133, CD 90, CD 44, NCAM, SOX 9, SOX 17, CK 18	SNA1L, vimentin, TGF-β, SHH genes, SHH/GLI, IL6/JAK2/STAT3	[54,70,71,72,73,74,75,76,77,78,79,80,81,82,83,84,85,86,87,88,89,90,91,92,93,94,95]

Abbreviations: HBV: hepatitis B virus; HCV: hepatitis C virus; NAFLD: nonalcoholic fatty liver disease; SHH: Sonic hedgehog; PTCH-1: Patched-1; GLI-1:glioma-associated oncogene-1; CDK: cyclin-dependent kinase; Hh-FOXM1-TPX2: Hh-Forkhead box-M1 (FOXM 1)-targeting protein for Xenopus kinesin-like protein 2 (TPX 2); FAK/PI3K/AKT: focal adhesion kinase/phosphoinositide 3-kinase/protein kinase B; CHSY1: chondroitin sulfate synthase 1; MMP: matrix metalloproteinase; TAM: tumor-associated macrophage; ROS: reactive oxygen species; EMT: epithelial–mesenchymal transition; SCUBE 1: signal peptide-CUB domain and EGF domain containing protein 1; SUMO: small ubiquitin-like modifier; ABCC 1: ATP binding cassette subfamily C member 1; TAP 1: transporter associated with antigen processing 1; NRF 2: nuclear factor (erythroid-derived)-like 2; ChK 1: checkpoint kinase 1; SOX: sex-determining region Y-box; IL6/JAK2/STAT 3: interleukin-6 Janus kinase 2 signal transducer and activator of transcription 3.

## References

[1] Toh M.R., Wong Y.E.T., Wong S.H., Ng A.W.T., Loo L.H., Chow P.K.H., Ngeow J. (2023). Global epidemiology and genetics of hepatocellular lar carcinoma. Gastroenterology.

[2] Li Q., Cao M., Lei L., Yang F., Li H., Yan X., He S., Zhang S., Teng Y., Xia C. (2022). Burden of liver cancer: From epidemiology to prevention. Chin. J. Cancer Res..

[3] Yang J.D., Hainaut P., Gores G.J., Amadou A., Plymoth A., Roberts L.R. (2019). A global view of hepatocellular carcinoma: Trends, risk, prevention and management. Nat. Rev. Gastroenterol. Hepatol..

[4] McGlynn K.A., Petrick J.L., El-Serag H.B. (2021). Epidemiology of hepatocellular carcinoma. Hepatology.

[5] International Agency for Research on Cancer (2018). GLOBOCAN 2018, Liver Cancer Global WHO Report.

[6] Park J.W., Chen M., Colombo M., Roberts L.R., Schwartz M., Chen P.J., Kudo M., Johnson P., Wagner S., Orsini L.S. (2015). Global patterns of hepatocellular carcinoma management from diagnosis to death: The BRIDGE Study. Liver Int..

[7] Forner A., Llovet J.M., Bruix J. (2012). Hepatocellular carcinoma. Lancet.

[8] Alberts C., Clifford G.M., GeorgesNegro F., Lesi O.A., Hutin Y.F., de Martel C. (2022). Worldwide prevalence of hepatitis B virus and hepatitis C virus among patients with cirrhosis at country, region, and global levels: A systematic review. Lancet Gastroenterol. Hepatol..

[9] Younossi Z.M., Wong G., Anstee Q.M., Henry L. (2023). The global burden of liver disease. Clin. Gastroenterol. Hepatol..

[10] Shah P.A., Patil R., Harrison S.A. (2023). NAFLD-related hepatocellular carcinoma: The growing challenge. Hepatology.

[11] Rumray H., Arnold M., Ferlay J., Lesi O., Cabasag C.J., Vignat J., Laversanne M., McGlynn K.A., Soerjomataram I. (2022). Global burden of primary liver cancer in 2020 and predictions to 2040. J. Hepatol..

[12] Brown Z.J., Tsilimigras D.I., Ruff S.M., Mohseni A., Kamel I.R., Cloyd J.M., Pawlik T.M. (2023). Management of hepatocellular carcinoma. JAMA Surg..

[13] Ingham P.W. (2022). Hedgehog signaling. Curr. Top. Dev. Biol..

[14] Jiang J. (2022). Hedgehog signaling mechanism and role in cancer. Semin. Cancer Biol..

[15] Jeng K.S., Chang C.F., Lin S.S. (2020). Sonic Hedgehog signaling in organogenesis, tumors, and tumor microenvironments. Int. J. Mol. Sci..

[16] Swiderska-Syn M., Xie G., Michelotti G.A., Jewell M.L., Premont R.T., Syn W.K., Diehl A.M. (2016). Hedgehog regulates yes-associated protein 1 in regenerating mouse liver. Hepatology.

[17] Michelotti G.A., Xie G., Swiderska M., Choi S.S., Karaca G., Krüger L., Premont R., Yang L., Syn W.-K., Metzger D. (2013). Smoothened is a master regulator of adult liver repair. J. Clin. Investig..

[18] Jeng K.S., Sheen I.S., Leu C.M., Tseng P.H., Chng C.F. (2020). The role of smoothened in cancer. Int. J. Mol. Sci..

[19] Scales S.J., de Sauvage F.J. (2009). Mechanisms of Hedgehog pathway activation in cancer and implications for therapy. Trends Pharmacol. Sci..

[20] Chung S.I., Moon H., Ju H.L., Cho K.J., Kim D.Y., Han K.H., Eun J.W., Nam S.W., Ribback S., Dombrowski F. (2016). Hepatic expression of sonic hedgehog induces liver fibrosis and promotes hepatocarcinogenesis in a transgenic mouse model. J. Hepatol..

[21] Sicklick J.K., Li Y.X., Jayaraman A., Kannangai R., Qi Y., Vivekanandan P., Ludlow J.W., Owzar K., Chen W., Torbenson M.S. (2005). Dysregulation of the hedgehog pathway in human hepatocarcinogenesis. Carcinogenesis.

[22] Adolphe C., Hetherington R., Ellis T., Wainwright B. (2006). Patched-1 functions as a gatekeeper by promoting cell cycle progression. Cancer Res..

[23] Barnes E.A., Kong M., Ollendorff V., Donoghue D.J. (2001). Patched-1 interacts with cycline B1 to regulate cell cycle progression. EMBO J..

[24] Chen X.L., Cheng Q.Y., She M.R., Wang Q., Huang X.H., Cao L.Q., Fu X.H., Chen J.S. (2010). Expression of sonic hedgehog signaling components in hepatocellular carcinoma and cyclopamine-induced apoptosis through Bcl-2 downregulation in vitro. Arch. Med. Res..

[25] Skoda A.M., Simovic D., Karin V., Kardum V., Vranic S., Serman L. (2018). The role of the hedgehog signaling in cancer: A compressive review. Bosn. J. Basic. Med. Sci..

[26] Jeng K.S., Jeng C.J., Jeng W.J., Sheen I.S., Li S., Leu C., Tsay Y., Chang C.F. (2019). Sonic Hedgehog [signaling pathway as a potential target to inhibit the progression of hepatocellular carcinoma (Review). Oncol. Lett..

[27] Huang S., He J., Zhang X., Bian Y., Yang L., Xie G., Zhang K., Tang W., Stelter A.A., Wang Q. (2006). Activation of the hedgehog pathway in human hepatocellular carcinomas. Carcinogenesis.

[28] Dugum M., Hanouneh I., McIntyre T., Pai R., Aucejo F., Eghtesad B., Zein N. (2016). Sonic hedgehog signaling in hepatocellular Carcinoma. A pilot study. Mol. Clin. Oncol..

[29] Jeng K.S., Sheen I.S., Jeng W.J., Lin C.C., Lin C.K., Su J.C., Yu M.C., Fang H.Y. (2013). High expression of patched homolog-1 messenger RNA and glioma-associated oncogene-1 messenger RNA of sonic hedgehog signaling pathway indicates a risk of postresection recurrence of hepatocellular carcinoma. Ann. Surg. Oncol..

[30] Lin M., Guo L.M., Liu H., Du J., Yang J., Zhang L.J., Zhang B. (2010). Nuclear accumulation of glioms-associated oncogene 2 protein and enhanced expression of forkhead-box transcription factor M1 protein in human hepatocellular carcinoma. Histol. Histopathol..

[31] Arzumanyan A., Sambandam V., Clayton M.M., Choi S.S., Xie G., Diehl A.M., Yu D.-Y., Feitelson M.A. (2012). Hedgehog signaling blockade delays hepatocarcinogenesis induced by hepatitis B virus X protein. Cancer Res..

[32] Pereira T.A., Witek R.P., Syn W.K., Choi S.S., Bradrick S., Karaca G.F., Agboula K.M., Jung Y., Omenetti A., Moylan C.A. (2010). Viral factors induce hedgehog pathway activation in humans with viral hepatitis, cirrhosis and hepatocellular carcinoma. Lab. Investig..

[33] Choi S.S., Bradrick S., Qiang G., Mostafavi A., Chaturvedi G., Weinman S.A., Diehl A.M., Jhaveri R. (2011). Up-regulation of hedgehog pathway is associated with cellular permissiveness for hepatitis C virus replication. Hepatology.

[34] Kwon H., Song K., Han C., Chen W., Wang Y., Dash S., Lim K., Wu T. (2016). Inhibition of hedgehog signaling ameliorates hepatic inflammation in mice with nonalcoholic fatty liver disease (NAFLD). Hepatology.

[35] Cai H., Li H., Li J., Li X., Li Y., Shi Y., Wang D. (2016). Sonic hedgehog signaling pathway mediates development of hepatocellular carcinoma. Tumor Biol..

[36] Cheng W.T., Xu K., Tian D.Y., Zhang Z.G., Liu L.J., Chen Y. (2009). Role of Hedgehog signaling pathway in proliferation and invasiveness of hepatocellular carcinoma cells. Int. J. Oncol..

[37] Philips G.M., Chan I.S., Swiderska M., Schroder V.T., Guy C., Karaca G.F., Moylan C., Venkatraman T., Feuerlein S., Syn W.K. (2011). Hedgehog signaling antagonist promotes regression of both liver fibrosis and hepatocellular carcinoma in a murine model of primary liver cancer. PLoS ONE.

[38] Pinter M., Sieghart W., Schmid M., Dauser B., Prager G., Dienes H.P., Trauner M., Peck-Radosavljevic M. (2013). Hedgehog inhibition reduces angiogenesis by downregulation of tumoral VEGF -A expression in hepatocellular carcinoma. United Eur. Gastroenterol. J.

[39] Jeng K.S., Jeng C.J., Jeng W.J., Sheen I.S., Chang C.F., Hsiau H.I., Hung Z.H., Yu M.C., Chang F.Y. (2015). Sonic hedgehog pathway inhibitor mitigates mouse hepatocellular carcinoma. Am. J. Surg..

[40] Wang Y., Wang H., Yan Z., Li G., Hu G., Zhang H., Huang D., Wang Y., Zhang X., Yan Y. (2020). The critical role of dysregulated Hh-FOXM1-TPX2 signaling in human hepatocellular carcinoma cell proliferation. Cell Commun. Signal..

[41] Ding J., Yang Y.Y., Li P.T., Ma Y., Zhang L., Zhou Y., Jin C., Li H.Y., Zhu Y.F., Liu X.P. (2024). TGF-beat 1/SMAD 3-driven GLI2 isoform expression contributes to aggressive phenotypes of hepatocellular carcinoma. Cancer Lett..

[42] Liu Z., Tu K., Wang Y., Yao B., Li Q., Wang L., Dou C., Liu Q., Zheng X. (2017). Hypoxia accelerates aggressiveness of hepatocellular carcinoma cells involving oxidative stress, epithelial-mesenchymal transition and non-canonical hedgehog signaling. Cell. Physiol. Biochem..

[43] Fan Y.H., Ding J., Nguyen S., Liu X.J., Xu G., Zhou H.Y., Duan N.N., Yang S.M., Zern M.A., Wu J. (2016). Aberrant hedgehog signaling is responsible for the highly invasive behavior of a subpopulation of hepatoma cells. Oncogene.

[44] Che L., Ren J., Yuan Y.H., Jia J., Di L.J., Song G.H., Yu J., Wang X.L. (2008). Expression of genes related to sonic hedgehog signaling in human hepatocellular carcinoma. Beijing Da Xue Xue Bao. Yi Xue Ban J. Peking Univ. Health Sci..

[45] Che L., Yuan Y.H., Jia J., Ren J. (2012). Activation of sonic hedgehog signaling pathway is an independent potential prognosis predictor in human hepatocellular carcinoma patients. Chin. J. Cancer Res..

[46] Ding J., Li H.Y., Zhang L., Zhou Y., Wu J. (2021). Hedgehog signaling, a critical pathway governing the development and progression of hepatocellular carcinoma. Cells.

[47] Chen B., Hu Z., Li B., Lin X., Luo Z., Hu Z. (2019). The expressions of Hedgehog and P13K-AKT pathway components correlate with invasion and metastasis in hepatocellular carcinoma. Int. J. Exp. Pathol..

[48] Li H.Y., Yin F.F., Li X.Y., Jia W.N., Ding J., Zhang L., Wang Z.H., Hu Q.Q., Zuo J.L., Jia L.H. (2021). Novel aptasensor–based assay of sonic hedgehog ligand for detection of portal vein invasion of hepatocellular carcinoma. Biosens. Bioelectron..

[49] Chen J.S., Huang X.H., Wang Q., Huang J.Q., Zhang L.J., Chen X.L., Lei J., Cheng Z.X. (2012). Sonic hedgehog signaling pathway induces cell migration and invasion through focal adhesion kinase/AKT signaling-mediated activation of matrix metalloproteinase (MMP)-2 and MMP-9 in liver cancer. Carcinogenesis.

[50] Wang Y.H., Sui X.M., Sui Y.N., Zhu Q.W., Yan K., Wang L.S., Wang F., Zhou J.H. (2015). BRD4 induces cell migration and invasion in HCC cells through MMP-2 and MMP-9 activation mediated by the Sonic hedgehog signaling pathway. Oncol. Lett..

[51] Liu S.J., Zang Y.W., Huang C.J., Liu Y.J. (2023). Downregulation of Rab 23 inhibits hepatocellular carcinoma by repressing SHH signaling pathway. Cancer Rep..

[52] Zhang L., Zhang B., You W., Li P., Kuang Y. (2020). Rab 23 promotes hepatocellular carcinoma cell migration via Rac1/TGF-beta signaling. Pathol. Oncol. Res..

[53] Orrù C., Szydlowska M., Taguchi K., Zavattari P., Perra A., Yamamoto M., Columbano A. (2018). Genetic inactivation of Nrf2 prevents clonal expansion of initiated cells in a nutritional mode of rat hepatocarcinogenesis. J. Hepatol..

[54] Leung H.W., Lau Y.E.T., Leung C.O.N., Lei M.M.L., Mok E.H.K., Ma V.W.S., Cho W.C.S., Ng I.O.L., Yun J.P., Cai S.H. (2020). NRF2/SHH signaling cascade promotes tumor-initiating cell lineage and drug resistance in hepatocellular carcinoma. Cancer Lett..

[55] Liu C.H., Lan C.T., Chou J.F., Tseng T.J., Liao W.C. (2017). CHSY 1 promotes aggressive phenotypes of hepatocellular carcinoma cells via activation of the hedgehog signaling pathway. Cancer Lett..

[56] Chang A.I., Schwertschkow A.H., Nolta J.A., Wu J. (2015). Involvement of mesenchymal stem cells in cancer progression and metastases. Curr. Cancer Drug Targets.

[57] Wang P., Song W., Li H., Wang C., Shi B., Guo W., Zhong L. (2015). Association between donor and recipient smoothened gene polymer phisms and the risk of hepatocellular carcinoma recurrence following orthotopic liver transplantation in a Han Chinese population. Tumor Biol..

[58] Della Corte C.M., Viscardi G., Papaccio F., Esposito G., Martini G., Ciardiello D., Martinello F. (2017). Implication of the Hedgehog pathway in hepatocellular carcinoma. World J. Gastroenterol..

[59] Neophytou C.M., Panagi M., Stylianopoulos T., Papageorgis P. (2021). The role of tumor microenvironment in cancer metastasis: Molecular mechanisms and therapeutic opportunities. Cancers.

[60] Petrelli A., Perra A., Cora D., Sulas P., Menegon S., Manca C., Migliore C., Kowalik M.A., Ledda-Columbano G.M., Giordano S. (2014). MicroRNA/gene profiling unveils early molecular changes and nuclear factor erythroid related factor 2(NRF2) activation in a rat model recapitulating human hepatocellular carcinoma (HCC). Hepatology.

[61] Zhao J., Li J., Chen J., Lin Z., Zhang B., Deng L., Chen G., Wang Y. (2022). CAFs-derived SCUBE 1 promotes malignancy and stemness through the Shh/Gli 1 pathway in hepatocellular carcinoma. J. Transl. Med..

[62] Petty A.J., Li A., Wang X., Dai R., Heyman B., Hsu D., Huang X., Yang Y. (2019). Hedgehog signaling promotes tumor associated macrophages polarization to suppress intratumoral CD 8+ T cell recruitment. J. Clin. Investig..

[63] Zhang C.Y., Jiang Z.M., Ma X.F., Li Y., Liu X.Z., Li L.L., Wu W.H., Wang T. (2019). Saikosaponin-d Inhibits the Hepatoma Cells and Enhances Chemosensitivity Through SENP5-Dependent Inhibition of Gli1 SUMOylation Under Hypoxia. Front. Pharmacol..

[64] Wang Q., Huang S., Yang L., Zhao L., Yin Y., Liu Z., Chen Z., Zhang H. (2008). Down-regulation of sonic hedgehog signaling pathway activity is involved in 5-fluorouracil-induced apoptosis and motility inhibition in Hep3B cells. Acta Biochem. Biophys. Sin..

[65] Chen X., Lingala S., Khoobyari S., Nolta J., Zern M.A., Wu J. (2011). Epithelial mesenchymal transition and hedgehog signaling activation are associated with chemoresistance and invasion of hepatoma subpopulations. J. Hepatol..

[66] Ding J., Zhou X.T., Zou H.Y., Wu J. (2017). Hedgehog signaling pathway affects the sensitivity of hepatoma cells to drug therapy through the ABCC1 transporter. Lab. Investig..

[67] Zhou X.T., Ding J., Li H.Y., Zuo J.L., Ge S.Y., Jia H.L., Wu J. (2020). Hedgehog signaling mediates drug resistance through targeting TAP1 in hepatocellular carcinoma. J. Cell Mol. Med..

[68] Wang S., Wang Y., Xun X., Zhang C., Xiang X., Cheng Q., Hu S., Li Z., Zhu J. (2020). hedgehog signaling promotes sorafenib resistance in hepatocellular carcinoma patient-derived organoids. J. Exp. Cancer Res..

[69] Chen Y.J., Lin C.P., Hsu M.L., Shieh H.R., Chao N.K., Chao K.S. (2011). Sonic hedgehog signaling protects human hepatocellular carcinoma cells against ionizing radiation in an autocrine manner. Int. J. Radiat. Oncol. Biol. Phys..

[70] Leonard J.M., Ye H., Wetmore C., Karnitz L.M. (2008). Sonic hedgehog signaling impaired ionizing radiation induced checkpoint activation and induces genomic instability. J. Cell Biol..

[71] Tsai C.L., Hsu F.M., Tzen K.Y., Liu W.L., Cheng A.L., Cheng J.C. (2015). Sonic Hedgehog inhibition as a strategy to augment radiosensitivity of hepatocellular carcinoma. J. Gastroenterol. Hepatol..

[72] Zhang L., Theise N., Chua M., Reid L.M. (2008). The stem cell niche of human livers: Symmetry between development and regeneration. Hepatology.

[73] Sato K., Marzioni M., Meng F., Trancis H., Glaser S., Alpini G. (2019). Ductular reaction in liver diseases: Pathological mechanisms and translational significances. Hepatology.

[74] Turner R., Lozoya O., Wang Y., Cardinale V., Gaudio E., Alpini G., Mendel G., Wauthierr E., Barbier C., Alvaro D. (2011). Human hepatic stem cell and maturational liver lineage biology. Hepatology.

[75] Rangwala F., Guy C.D., Lu J., Suzuki A., Burchette J.L., Abdelmalek M.F., Chen W., Diehl A.M. (2011). Increased production of sonic hedgehog by ballooned hepatocytes. J. Pathol..

[76] Xu G., Ye J., Liu X.J., Zhang N.P., Zhao Y.M., Fan J., Liu X.P., Wu J. (2017). Activation of pluripotent genes in hepatic progenitor cells in the transition of nonalcoholic steatohepatitis to pre-malignant lesions. Lab. Investig..

[77] Plaks V., Kong N., Werb Z. (2015). The cancer stem cell niche: How essential is the niche in regulating stemness of tumor cells?. Cell Stem Cell.

[78] Fan Q.M., Jing Y.Y., Yu G.F., Kou X.R., Ye F., Gao L., Li R., Zhao Q.D., Yang Y., Lu Z.H. (2014). Tumor-associated macrophages promote cancer stem cell-like properties visa transforming growth factor-beta-1 induced epithelial-mesenchymal transition in hepatocellular carcinoma. Cancer Lett..

[79] Duan H., Liu Y., Gao Z., Huang W. (2020). Recent advances in drug delivery systems for targeting cancer stem cells. Acta Pharm. Sin. B.

[80] Ma S., Chan K.-W., Hu L., Lee T.K.-W., Wo J.Y.-H., Ng I.O.-L., Zheng B.-J., Guan X.-Y. (2007). Identification and characterization of tumorigenic liver cancer stem/progenitor cells. Gastroenterology.

[81] Gramantieri L., Giovannini C., Suzzi F., Leoni I., Fornari F. (2021). Hepatic cancer stem cells: Molecular mechanisms, therapeutic implications, and circulating biomarkers. Cancers.

[82] Sukowati C., Anfuso B., Pascut D., Tiribelli C. (2015). Multidrug resistance in hepatic cancer stem cells: The emerging role of miRNAs. Expert Rev. Gastroenterol. Hepatol..

[83] Jeng K.S., Chang C.F., Sheen I.S., Jeng C.J., Wang C.H. (2023). Cellular and molecular biology of cancer stem cells of hepatocellular carcinoma. Int. J. Mol. Sci..

[84] Sukowati C.H.C. (2019). Heterogeneity of hepatic cancer stem cells. Adv. Exp. Med. Biol..

[85] Li Z. (2013). CD133, A stem cell biomarker and beyond. Exp. Hematol. Oncol..

[86] Liu Y.C., Yeh C.T., Lin K.H. (2020). Cancer stem cell functions in hepatocellular carcinoma and comprehensive therapeutic strategies. Cells.

[87] Yang Z.F., Ngai P., Ho D.W., Yu W.C., Ng M.N., Lau C.K., Li M.L.Y., Tam K.H., Lam C.T., Poon R.T.P. (2008). Identification of local and circulating cancer stem cells in human liver cancer. Hepatology.

[88] Lingala S., Cui Y.Y., Chen X., Ruebner B.H., Qian X.F., Zern M.A., Wu J. (2010). Immunohistochemical staining of cancer stem cell markers in hepatocellular carcinoma. Exp. Mol. Pathol..

[89] Zarebska I., Gzil A., Dur´slewicz J., Jaworski D., Antosik P., Ahmadi N., ´Switała M.S., Grzanka D., Szylberg Ł. (2021). The clinical, prognostic and therapeutic significance of liver cancer stem cells and their markers. Clin. Res. Hepatol. Gastroenterol..

[90] Suetsugu A., Nagaki M., Aoki H., Motohashi T., Kunisada T., Moriwaki H. (2006). Characterization of CD133+ hepatocellular carcinoma cells as cancer stem/progenitor cells. Biochem. Biophys. Res. Commun..

[91] Yin S., Li J., Hu C., Chen X., Yao M., Yan M., Jiang G., Ge C., Xie H., Wan D. (2007). CD133 positive hepatocellular carcinoma cells possess high capacity for tumorigenicity. Int. J. Cancer.

[92] Song W., Li H., Tao K., Li R., Song Z., Zhao Q., Zhang F., Dou K. (2008). Expression and clinical significance of the stem cell marker CD133 in hepatocellular carcinoma. Int. J. Clin. Pract..

[93] Yamashita T., Toida M., Kato K., Long N.K., Miyazaki Y., Asaka Y., Hatakeyama D., Yonemoto K., Makita H., Kato Y. (2009). Cytoplasmic expression of CD133 is an important risk factor for overall survival in hepatocellular carcinoma. Oncol. Rep..

[94] Kim B.H., Park J.-W., Kim J.S., Lee S.-K., Hong E.K. (2019). Stem cell markers predict the response to Sorafenib in patients with hepatocellular carcinoma. Gut Liver.

[95] Zhang K., Che S., Pan C., Su Z., Zheng S., Yang S., Zhang H., Li W., Wang W., Liu J. (2018). The SHH/Gli axis regulates CD 90-mediated liver cancer stem cell function by activating the IL6/JAK2 pathway. J. Cell. Mol. Med..

[96] Tripathy A., Thakurela S., Sahu M.K., Uthansingh K., Singh A., Narayan J., Ajay A.K., Singh V., Kumari R. (2020). Fatty changes associated with N-nitrosodiethylamine (DEN) induced hepatocellular carcinoma: A role of sonic hedgehog signaling pathway. Genes Cancer.

[97] Syn W.K., Jung Y., Omenetti A., Abdelmalek M., Guy C.D., Yang L., Wang J., Witek R.P., Fearing C.M., Pereira T.A. (2009). Hedgehog-mediated epithelial-to-mesenchymal transition and fibrogenic repair in nonalcoholic fatty liver disease. Gastroenterology.

[98] Bhatia B., Hsieh M., Kenney A.M., Nahle Z. (2011). Mitogenic sonic hedgehog signaling drives E2F1-dependent lipogenesis in progenitor cells and medulloblastoma. Oncogene.

[99] Wang S.N., Yang S.F., Tsai H.H., Lee K.T., Yeh Y.T. (2014). Increased adiponectin associated with poor survival in hepatocellular carcinoma. J. Gastroenterol..

[100] Siegel A.B., Goyal A., Salomao M., Wang S., Lee V., Hsu C., Rodriguez R., Hershman D.L., Brown R.S., Neugut A.I. (2015). Serum adiponectin is associated with worsened overall survival in prospective cohort of hepatocellular carcinoma patients. Oncology.

[101] Zhu J., Wu J., Frizell E., Liu S.I., Bashey R., Rubin R., Norton P., Zern M.A. (1999). Rapamycin inhibits hepatic srellate cell proliferation in vitro and limits fibrogenesis in an in vivo model of liver fibrosis. Gastroenterology.

[102] Kitamura Y., Umemura K., Kanki K., Kodama Y., Saito K., Itoh K., Yamamoto M., Masegi T., Nishikawa A., Hirose M. (2007). Increased susceptibility to hepatocarcinogenicity of Nrf2-deficient mice exposed to 2-amino-3-methylimidazo [4,5-f] quinolone. Cancer Sci..

[103] Sporn M.B., Liby K.T. (2012). NRF2 and cancer: The good, the bad and the importance of context. Nat. Rev. Cancer.

[104] Guichard C., Amaddeo G., Imbeaud S., Ladeiro Y., Pelletier L., Maad I.B., Calderaro J., Bioulac-Sage P., Letexier M., Degos F. (2012). Integrated analysis of somatic mutations and focal copy-number changes identifies key genes and pathways in hepatocellular carcinoma. Nat. Genet..

[105] Jeng K.S., Sheen I.S., Jeng W.J., Yu M.C., Hsiau H.I., Chang F.Y., Tsai H.H. (2013). Activation of the sonic hedgehog signaling pathway occurs in the CD133 positive cells of mouse liver cancer Hepa 1–6 cells. Onco Targets Ther..

[106] Sari I.N., Phi L.T.H., Jun N., Wijaya Y.T., Lee S., Kwon H.Y. (2018). Hedgehog signaling in cancer: A prospective therapeutic target for eradicating cancer stem cells. Cells.

[107] Jeng K.S., Sheen I.S., Jeng W.J., Yu M.C., Tsai H.H., Chang F.Y., Su J.C. (2012). Blockade of the sonic hedgehog pathway effectively inhibits the growth of hepatoma in mice: An in vivo study. Oncol. Lett..

[108] Sheng X., Sun X., Sun K., Sui H., Qin J., Li Q. (2016). Inhibitory effect of bufalin combined with hedgehog signaling pathway inhibitors on proliferation and invasion and metastasis of liver cancer cells. Int. Oncol..

[109] Kim Y., Yoon J.W., Xiao X., Dean N.M., Monia B.P., Marcusson E.G. (2007). Selective down-regulation of glioma-associated oncogene 2 inhibits the proliferation of hepatocellular carcinoma cells. Cancer Res..

[110] Sun C., Zhang Z., He P., Zhou Y., Xie X. (2017). Involvement of P13K/Akt pathway in the inhibition of hepatocarcinoma cell invasion and metastasis induced by SASH through downregulating Shh-Gli-1 signaling. Int. J. Biochem. Cell Biol..

[111] Kumar V., Sethi B., Staller D.W., Xin X., Ma J., Dong Y., Talmon G.A., Mahato R.I. (2023). Anti-miR-96 and Hh pathway inhibitor MDB5 synergistically ameliorate alcohol-associated liver injury in mice. Biomaterials.

[112] Hong E., Jayachadran P., Brewster R. (2010). The polarity protein Pard3 is required for centrosome positioning during neurulation. Dev. Biol..

[113] Wu J., Tan H.Y., Chan Y.T., Feng Z., Yuan H., Zhang C., Feng Y., Wang N. (2024). PARD3 drives tumorigenesis through activating sonic hedgehog signaling in tumour-initiating cells in liver cancer. J. Exp. Clin. Cancer Res..

[114] Harada K., Ohashi R., Naito K., Kanki K. (2020). Hedgehog signal inhibitor GANT 61 inhibits the malignant behavior of undifferentiated hepatocellular carcinoma cells by targeting non-canonical GLI signaling. Int. J. Mol. Sci..

[115] Torbenson M., McCabe C.E., O’Brien D.R., Yin J., Bainter T., Tran N.H., Yasir S., Chen Z.E., Dhanase K.R., Ahn K.S. (2022). Morphological heterogeneity in beta-catenin mutated hepatocellular carcinoma: Implications for tumor molecular classification. Hum. Pathol..

[116] Shin S.H., Park J.Y., Hwang C., Lee H.J., Shin D.H., Kim J.Y., Ryu J.H., Yang K.H., Lee T.B., Lee J.H. (2023). Histological subtypes of hepatocellular carcinoma: Their clinical and prognostic significance. Ann. Diagn. Pathol..

[117] Bioulac-Sage P., Gouw A.S.H., Balabaud C., Sempoux C. (2022). Hepatocellular adenoma: What we know, what we do not know, and why it matters. Histopathology.

[118] Tse J.R., Felker E.R., Cao J.J., Liang T., Lu D.S.K., Raman S.S. (2023). Hepatocellular adenoma subtypes based on 2017 Classification System: Exploratory study of Gadoxetate Disodium-enhanced MRI features with proposal of a diagnostic algorithm. Am. J. Roentgenol..

[119] Ducatel A., Trillaud H., Reizine E., Vilgrain V., Sempoux C., Schmidt-Kobbe S., Gouw A.S.H., de Haas R.J., Julien C., Paradis V. (2023). Sonic hedgehog hepatocellular adenoma: Magnetic resonance imaging features and correlation with histology. Eur. Radiol..

[120] Nault J.C., Couchy G., Balabaud C., Morcrette G., Caruso S., Blanc J.F., Bacq Y., Calderaro J., Paradis V., Ramos J. (2017). Molecular classification of hepatocellular adenoma associates with risk factors, bleeding, and malignant transformation. Gastroenterology.

